# Responsibility of Individuals and Stakeholders for Obesity and a Healthy Diet: Results From a German Survey

**DOI:** 10.3389/fpsyt.2020.00616

**Published:** 2020-07-03

**Authors:** Sandra Bayer, Theresa Drabsch, Gunther Schauberger, Hans Hauner, Christina Holzapfel

**Affiliations:** ^1^ School of Medicine, Institute for Nutritional Medicine, Technical University of Munich, Munich, Germany; ^2^ Chair of Epidemiology, Department of Sport and Health Sciences, Technical University of Munich, Munich, Germany; ^3^ ZIEL – Institute for Food & Health, Technical University of Munich, Freising, Germany

**Keywords:** healthy diet, responsibility, political approaches, obesity, survey, Germany

## Abstract

**Introduction:**

Overweight and obesity are thought to be mainly caused by an energy-rich diet and a sedentary lifestyle. The opinions of those with and without obesity about an individual´s and stakeholder´s responsibility for overweight and obesity as well as a healthy diet is rather unclear. Therefore, a survey was conducted to assess the thoughts of persons with and without obesity about the responsibilities for a high body weight and healthy diet.

**Methods:**

This telephone-based survey was conducted in Germany. Landline and mobile phone users older than 17 years were quota sampled to represent the German population (n=1,003). Additionally, 354 adults with a body mass index (BMI) ≥ 30 kg/m^2^ were included in the survey population. Questions on weight management, eating and drinking and anthropometry were asked. Furthermore, the opinions of participants on the responsibility of individuals and stakeholders for obesity and a healthy diet were collected. Data was statistically weighted by age, gender, education, domicile, and BMI.

**Results:**

Data of 1,357 persons (51.1% female, age: 50.5 ± 18.5 years, 15.9% with BMI ≥ 30 kg/m^2^) were analyzed. Participants responded that the general causes of a high body weight were low physical activity (82.7%) and excessive caloric intake (80.5%) followed by a lack of will power (72.1%). Almost 90% of the survey population reported that each individual is responsible for his/her own healthy diet. More than 85% of the survey population agreed that a healthy diet in kindergarten and nutrition education at schools should be the preferred approaches when politics take care of a person´s healthy diet. Sub-analyses revealed that BMI, sex, age, and education are potential confounders.

**Conclusion:**

This German survey showed that the majority of participants indicated that the responsibility for a healthy diet lies with the individual and high body weight is caused by self-controlled attitudes. These results suggest that the survey population underestimates societal and environmental factors that contribute to the development of obesity, which could lead to attitudes that facilitate weight-related stigmatization. Furthermore, survey participants indicated that they would support policy-driven measures that promote a healthy diet.

## Introduction****

The increasing prevalence of obesity has become a global burden (1–3). While in 1980 about 851 million people were overweight or obese, the number (2.1 billion) is more than double in 2013 (2). In Germany, the prevalence of overweight (67.1% men, 53.0% women) has been nearly stable over the last 20 years. However, the number of adults (4) as well as children and adolescents (2) with obesity continued to increase during this time. Obesity is a major modifiable determinant in the development of many non-communicable diseases (NCDs) (5, 6). In 2016, 71% of global deaths were estimated to be caused by NCDs (7). Therefore, more efforts to prevent obesity and to facilitate a healthy lifestyle are needed to significantly reduce the number of global deaths due to NCDs (6).

Obesity is mostly driven by an excess of caloric intake and a sedentary lifestyle (5). Prevention strategies have mainly focused on the individual level (8). However, the development of obesity is complex and is also caused by the current obesogenic environment (5, 9). A literature review concluded that there is an interaction between the obesogenic environment and the individual lifestyle (10). The World Health Organization (WHO) stated that the burden of obesity can only be reduced when individuals have access to an environment that facilitates a healthy lifestyle (5). Hence, the WHO initiated a Global Action Plan for the Prevention and Control of Non-communicable Diseases to decrease the prevalence of NCDs by 25% worldwide until 2025 (11). This should be achieved by implementing prevention strategies on both the individual and the population level.

Studies have shown that the stigmatization of people with obesity is frequent and a major concern (12–14). One reason for this is that the public generally does not view obesity as a medical condition, whereas experts consider obesity to be a chronic disease (15). The media and the public often communicate that obesity is the result of a loss of self-control, leading to high energy intake and lack of physical activity. This misconception promotes the stigmatization of people with overweight and obesity and impedes their adequate care. Besides, a policymaker survey from the European Association for the Study of Obesity (EASO) explored the extent to which policymakers recognize their responsibility in reducing the prevalence of obesity (16). Policymakers answered that individuals, families and the food industry are most responsible for tackling obesity in the future (16). Addressing stigmatization and discrimination as well as the responsibility of policymakers to change the obesogenic environment are therefore rarely on the agenda. Increased attention on the misconceptions regarding the origin of obesity is warranted. Stakeholders and policymakers should be encouraged to develop a plan that facilitates adopting a healthy lifestyle in all stages of life by promoting preventive and therapeutic strategies that are free and accessible to all. To achieve these goals, more data from the general population are needed.

The opinion of those with and without obesity regarding the reasons for developing overweight and obesity is rather unclear. Likewise, a focus group study explored beliefs from the general population regarding the individual and stakeholder’s responsibility to promote a healthy diet and to prevent high body weight (17). Therefore, the aim of this representative, as well as target group-specific survey was to collect data on the beliefs and perceptions from a representative sample of the German population about the individual versus societal responsibilities regarding obesity development and a healthy diet.

## Methods

### Sample Characteristics

Between January and March 2019, a nationwide telephone-based survey was conducted in Germany by USUMA GmbH, a social research and market analysis company. A scientific Random Digit Dialing (RDD) method (ADM-Sampling System, Dual Frame) was used to randomly sample landline and mobile phone users who were at least 18 years old. The used RDD sampling frame followed a further developed version of the Gabler-Häder procedure, which allowed potential participants who were not listed in official registers to be contacted. About 40% of the initial sample were mobile numbers, as 15% of the German population do not have access to landline connections. For the random selection of a participant within one household, the Kish selection grid was used (USUMA Markt- und Sozialforschung, Berlin, Germany).

For the representative sample, 2,361 subjects were contacted. A response rate of 42.5% (N = 1,003) was achieved. To compare the results between persons with a BMI < 30.0 kg/m^2^ and persons with a BMI ≥ 30.0 kg/m^2^, target group-specific interviews for persons with obesity (N = 354) were added. This sampling was complementary to the representative sample. In total, data from 1,357 subjects were collected, of whom 505 participants had a BMI ≥ 30.0 kg/m^2^. Each interview took about 25 minutes and was conducted by trained staff. Due to the performance of the survey by a professional provider, this survey was not approved by the local ethical review committee. Participants provided their oral informed consent before participation, under the premise that participation in this survey was voluntary and analyses are conducted anonymously. Oral consent is common in representative survey research in Germany to avoid bias through non-response.

### Development of the Questionnaire

The survey team developed a standardized survey-specific questionnaire based on the ideas collected in two focus groups (BMI < 30.0 kg/m^2^, BMI ≥ 30.0 kg/m^2^). Before finalizing the questionnaire, it was pretested in paper form and evaluated in terms of understanding and length by 30 computer-assistant telephone-based interviews (CATI) by trained staff.

At the beginning of the interview, each person was informed about the research project, voluntary participation and data protection. Data about sociodemographic variables (age, gender, marital status, education, occupation, migration background) were collected by 10 questions. BMI was calculated according to the WHO criteria (18), using self-reported anthropometric data (height, weight) (4 questions). As BMI was a major parameter for the sub-analyses, a computer-based method was implemented, which allowed to calculate the BMI without naming the actual weight. First, body height, which was mandatory for participation, was asked. By reluctance to name the body height, the person was excluded and the interview has been stopped. After mentioning body height, the participant was asked for his body weight to calculate the BMI with the equation BMI = weight in kilogram/(height in meters)^2^. In case of reluctance to report body weight, the interviewer asked for pre-calculated weight ranges that allowed to calculate the participant´s BMI according to the WHO criteria (underweight/normal weight: BMI < 25.0 kg/m^2^, overweight: BMI 25.0–29.9 kg/m^2^, obesity: BMI ≥ 30.0 kg/m^2^). This method was applied by five participants who did not report their body weight. In total, 42 questions were asked. The main part of the questionnaire was divided into four topics - weight management (4 questions), nutritional questions (2 questions: dietary habits, 1 question: responsibility, 1 question: political approaches), state of knowledge of personalized genotype-based dietary recommendations (6 questions) and opinions and expectations of personalized genotype-based dietary recommendations (14 questions).

Screening questions were used to tailor the questionnaire to individual participants. For example, if participants responded to certain questions with “no” or “I do not know”, some related questions were skipped. The questionnaire consisted of open-ended, semi-closed and closed questions with single and multi-choice answer options. Several questions had responses based on a five-point Likert scale (e.g. 1=not important to 5=very important). Participants were also given the choice of responding with “no answer” to decrease response bias. The present analysis was focused on the responsibility of individuals and stakeholders for a high body weight and a healthy diet.

### Data Analysis

Before analyzing, data were statistically weighted by age, gender, education and domicile according to the population demographics by iterative proportional fitting (19). Furthermore, the percental distribution of persons with BMI < 30.0 kg/m^2^ and ≥ 30.0 kg/m^2^ of the total sample (N=1,357) was proportioned to represent the percental distribution of persons with BMI < 30.0 kg/m^2^ and ≥ 30.0 kg/m^2^ of the initial sample (N=1,003). Therefore, 852 persons with a BMI < 30.0 kg/m^2^ represent 1,141 (84.1%) persons and 505 persons with a BMI ≥ 30.0 kg/m^2^ represent 216 (15.9%) persons within the total sample population (1,357 interviews). Due to this, the percental distribution of the BMI categories was according to the population demographics and no further iterative proportional fitting was done. The different variables within each question were randomly chosen for each survey participant to avoid order bias. Because of screening questions, the number of questions per participant was varying. Missing values including answers with “no answer” or “I do not know” were eliminated listwise in the analysis. For the present work, 16 questions were used for the statistical analysis. The results of the other questions are published elsewhere (Bayer S et al., submitted). The statistical software program SPSS (SPSS version 25.0, SPSS Inc., Chicago, IL, USA) was used for the descriptive statistics (frequencies and percentages). Analyses were performed for the total sample as well as for subgroups such as BMI (BMI < 30.0 kg/m^2^, BMI ≥ 30.0 kg/m^2^), gender, age, and education, because subgroup differences were assumed. Furthermore, in the statistical software R (20) weighted Chi-squared independence tests were performed to compare the respective subgroups. In cases, where the proportions of three answer categories were compared, the test refers to the comparison of categories (1, 2) vs. 3. For each test, the corresponding p-values for the null hypothesis of equal proportions among all subgroups was reported. Data are shown as numbers and percentages of the total sample.

## Results

### Characteristics of the Participants

In **Table 1**, the characteristics of the survey population are summarized. In total, 1,357 people participated in the survey, of whom 51.1% (694/1,357) were female, 53.0% (718/1,353) were married and 37.7% (482/1,279) reported having a higher education entrance qualification. The mean age was 50.5 ± 18.5 years. Half of the survey population was employed (726/1,356) and 18.5% (243/1,311) had a migration background (**Table 1**). Respondents reported a calorie-reduced diet (55.3%, 401/725), a low-carb diet (49.4%, 358/725) and a low-fat diet (48.1%, 349/725) as weight loss strategies most often tried (**Table 2**). About 77% (77.3%, 1,048/1,355) of the survey population reported that what they eat and drink is important to them (**Table 3**).

**Table 1:** Characteristics of the survey population.

Variable	Number	
	n/N^#^	%
*Gender*		
Female	694/1,357	51.1
Male	663/1,357	48.9
*Age (years)*		
18-35	345/1,357	25.5
36-65	690/1,357	50.8
>65	322/1,357	23.7
*BMI (kg/m^2^)*		
<18.5	19/1,357	1.4
18.5-24.9	662/1,357	48.8
25-29.9	459/1,357	33.8
≥30	216/1,357	15.9
*Marital status*		
Single	415/1,353	30.7
Married	718/1,353	53.0
Divorced/widowed	220/1,353	16.3
*Education (years)^1^*		
Student	3/1,279	0.2
8/9	360/1,279	28.1
10	424/1,279	33.2
12/13	482/1,279	37.7
No education	10/1,279	0.8
*Occupation*	726/1,356	53.6
*Immigrant background*	243/1,311	18.5

^1^What is your highest level of education? Possible answers: still studying, certificate of secondary education (8/9 years), a general certificate of secondary education (10 years), higher education entrance certification (12/13 years), no student/education, no answer.

^#^Persons with answers “no answer” are not included in statistical analysis. The number of those answers can be calculated by the difference between the total population (N=1,357) and the number of answers given for each variable.

BMI, body mass index; data is statistically weighted by age, gender, education, domicile and BMI.

**Table 2:** Weight loss strategies of participants (N=745).

Variable*^1^* ^†^*	Total		BMI (kg/m^2^)			Gender			Age (years)				Education (years)				
			< 30	≥ 30	p	Women	Men	p	18–35	36–65	> 65	p	Student/none	8/9	10	12/13	p
	n^#^	%	%	%		%	%		%	%	%		%	%	%	%	
Increased physical activity/sport	547	75.4	77.5	69.4	0.053	75.6	75.3	0.635	78.2	77.1	60.5	<0.000	42.9	71.1	73.3	74.9	0.269
Calorie reduced diet	401	55.3	50.0	71.0	<0.000	59.7	49.5	0.001	55.8	51.6	57.6	0.322	85.7	58.7	48.3	52.4	0.022
Low carb diet	358	49.4	45.2	61.7	<0.000	52.1	46.0	0.036	58.8	42.5	50.5	0.001	57.1	56.7	41.1	47.6	0.003
Low fat diet	349	48.1	42.3	66.1	<0.000	51.6	45.7	0.114	43.0	44.0	57.6	0.002	85.7	54.2	46.2	41.6	0.002
High protein diet	271	37.4	32.5	51.9	<0.000	40.3	33.7	0.020	40.6	34.6	36.6	0.388	85.7	37.3	35.2	34.8	0.017
Food combining	112	15.4	14.6	18.0	0.181	18.6	11.4	0.002	14.5	14.3	18.0	0.451	0.0	15.9	18.2	10.5	0.025
Something else	80	11.0	7.6	21.3	<0.000	10.5	11.1	0.753	9.7	10.1	15.1	0.352	0.0	5.5	12.7	23.2	0.042
Surgery/drugs	67	9.2	7.7	13.4	0.010	10.3	8.3	0.249	8.5	6.6	15.1	0.001	0.0	9.5	13.1	4.5	0.001
Weight Watchers^®^	55	7.6	5.2	14.8	<0.000	11.2	2.5	<0.000	5.5	6.1	12.2	0.012	0.0	9.5	6.4	7.9	0.444
Program at a medical centre	53	7.3	4.4	15.8	<0.000	6.6	8.3	0.457	4.2	7.1	9.9	0.079	57.1	9.0	8.5	4.1	<0.000
Genotype-based diet	33	4.6	4.2	5.5	0.368	6.1	2.5	0.007	7.3	3.9	2.9	0.081	0.0	9.5	2.1	3.4	0.001

^1^Which strategy have you used to lose body weight? Possible answers: yes, no, none, no answer; Shown is “yes” as answer.

^†^Screening question, which means that only persons who tried to lose weight got this question.

*Multiple answers possible.

^#^Persons with answers “no answer” or “none” are not included in statistical analysis. The number of those answers can be calculated by the difference between the participants, who got the question (N=745), and the number of answers given for each variable.

BMI, body mass index; p, p-value; data is statistically weighted by age, gender, education, domicile and BMI.

**Table 3:** Importance of eating and drinking.

Variable	Answer^†^	Total		BMI (kg/m^2^)			Gender			Age (years)				Education (years)				
				< 30	≥ 30	p	Women	Men	p	18–35	36–65	> 65	p	Student/none	8/9	10	12/13	p
		n/N^#^	%	%	%		%	%		%	%	%		%	%	%	%	
*Importance of what you eat and drink^1^*	1	52/1,355	3.8	3.3	7.0		4.6	3.0		3.2	3.8	4.7		0.0	4.2	4.5	3.1	
	2	255/1,355	18.8	16.9	28.8		15.4	22.5		24.6	15.1	15.7		17.6	22.6	18.8	16.3	
	3	1,048/1,355	77.3	79.8	64.2	<0.000	80.0	74.5	0.014	72.2	81.2	74.7	0.001	76.9	73.3	76.7	80.6	0.086
*Importance of facts for eating and drinking^2^*																		
Food rich in fibre (e.g. whole grain products)	1	219/1,354	16.2	15.1	21.5		11.7	20.9		26.2	13.8	10.6		38.5	17.8	14.9	16.1	
	2	330/1,354	24.4	23.8	27.6		24.7	24.1		28.2	24.5	19.9		15.4	24.0	23.6	25.7	
	3	805/1,354	59.5	61.1	50.9	0.005	63.6	55.1	0.001	45.6	61.7	69.5	<0.000	38.5	58.2	61.5	58.2	0.358
Self-prepared and fresh meals (e.g. no convenience products)	1	129/1,350	9.6	9.0	13.2		7.1	12.2		10.4	9.2	9.7		53.8	9.2	9.5	9.1	
	2	193/1,350	14.3	14.1	15.6		8.5	20.4		21.7	12.8	9.1		0.0	12.8	14.7	14.9	
	3	1,028/1,350	76.1	77.0	72.1	0.074	84.4	67.4	<0.000	67.8	78.1	81.1	<0.000	46.2	77.9	75.8	75.9	0.115
Adequate fluids	1	92/1,356	6.8	6.4	9.2		5.6	8.0		8.4	6.7	5.3		23.1	7.5	6.4	6.7	
	2	152/1,356	11.2	11.0	12.4		11.3	11.2		16.8	8.6	10.9		0.0	13.3	11.1	11.2	
	3	1,112/1,356	82.0	82.6	78.3	0.144	83.1	80.8	0.283	74.9	84.6	83.8	<0.000	76.9	79.2	82.5	82.1	0.567
Small portions	1	306/1,344	22.8	22.8	22.8		15.8	29.7		41.4	17.5	13.5		69.2	18.1	23.2	26.4	
	2	478/1,344	35.6	35.6	35.3		35.5	35.8		35.9	35.9	33.7		7.7	34.8	36.0	38.7	
	3	560/1,344	41.7	41.6	41.9	0.960	48.7	34.5	<0.000	22.6	46.2	52.9	<0.000	23.1	47.1	40.8	34.9	0.003
Balanced and healthy diet	1	90/1,357	6.6	5.3	13.4		4.2	9.2		10.1	5.8	4.0		30.8	7.5	6.6	6.0	
	2	185/1,357	13.6	11.6	24.4		10.2	17.2		18.3	12.6	11.2		46.2	13.9	14.9	11.0	
	3	1,082/1,357	79.7	83.1	62.2	<0.000	85.6	73.6	<0.000	71.6	81.6	84.7	<0.000	30.8	78.6	78.5	83.0	<0.000
Regional products	1	172/1,351	12.7	11.8	18.0		9.7	15.8		25.7	8.4	7.9		46.2	12.3	12.3	12.7	
	2	257/1,351	19.0	18.9	19.4		17.1	21.1		21.4	18.8	17.0		23.1	19.2	17.3	21.2	
	3	922/1,351	68.2	69.3	62.7	0.058	73.2	63.1	<0.000	52.9	72.9	75.1	<0.000	30.8	68.5	70.4	66.1	0.016
Simple and fast food (e.g. fast food, frozen products, currywurst)	1	925/1,352	68.4	69.3	63.9		75.7	60.8		61.7	68.1	76.3		7.7	64.9	73.1	77.4	
	2	238/1,352	17.6	17.8	16.7		13.4	22.0		16.8	19.4	14.6		38.5	17.8	15.1	22.6	
	3	189/1,352	14.0	12.9	19.4	0.014	10.9	17.2	<0.000	21.4	12.5	9.2	<0.000	53.8	17.3	11.8	13.7	<0.000
Well tasty food	1	52/1,353	3.8	3.7	4.6		3.9	3.8		5.8	2.5	5.0		0.0	3.6	5.0	3.3	
	2	137/1,353	10.1	10.6	7.4		9.1	11.2		9.8	10.9	9.0		0.0	11.4	7.5	11.6	
	3	1,164/1,353	86.0	85.7	88.0	0.337	87.0	85.0	0.313	84.4	86.6	86.0	0.651	100.0	85.0	87.5	85.0	0.294
Adequate fruit and vegetable intake (5 a day)	1	147/1,352	10.9	9.8	16.7		6.4	15.6		13.9	11.8	6.3		38.5	11.9	11.3	9.8	
	2	263/1,352	19.5	18.8	23.1		15.5	23.6		22.9	20.5	13.4		38.5	21.1	20.5	17.5	
	3	943/1,352	69.7	71.5	60.2	0.001	78.1	60.8	<0.000	63.5	67.9	80.6	<0.000	23.1	66.9	68.2	72.7	0.001
Big portions	1	689/1,352	51.0	51.0	50.5		59.5	42.1		39.6	52.2	60.7		38.5	52.4	47.6	53.6	
	2	414/1,352	30.6	31.0	27.8		27.1	34.3		35.3	30.8	25.5		23.1	25.6	36.5	29.3	
	3	249/1,352	18.4	17.9	20.8	0.325	13.5	23.6	<0.000	25.1	17.1	20.2	<0.000	38.5	22.0	15.9	17.0	0.017
Vegetarian/vegan food	1	883/1,348	65.5	63.6	75.1		60.3	70.8		63.8	65.4	67.9		81.8	70.3	67.8	59.3	
	2	234/1,348	17.4	18.1	13.8		19.7	15.0		14.2	17.9	19.7		0.0	18.3	15.6	18.7	
	3	231/1,348	17.1	18.3	11.1	0.007	20.0	14.2	0.005	22.3	29.6	12.4	0.004	18.2	11.4	16.5	22.0	<0.000
On my mental state based food	1	258/1,334	19.3	10.2	19.2		12.9	26.0		30.0	13.7	19.8		46.2	14.5	21.3	20.4	
	2	320/1,334	24.0	23.1	28.5		23.8	24.0		25.4	25.0	20.1		15.4	24.5	26.1	21.4	
	3	756/1,334	56.7	57.5	52.3	0.148	63.3	50.0	<0.000	44.6	61.3	60.1	<0.000	38.5	61.0	52.6	58.2	0.059
Food high in protein	1	289/1,338	21.6	21.5	21.5		20.2	22.9		22.7	23.8	15.7		23.1	18.8	21.4	23.9	
	2	568/1,338	42.5	43.1	39.3		40.7	44.2		37.2	44.9	42.9		15.4	46.6	43.0	39.6	
	3	481/1,338	36.0	35.4	39.4	0.290	39.0	32.9	0.021	40.1	31.2	41.3	0.002	53.8	34.6	35.6	36.5	0.403
Avoidance of sugar-sweetened beverages (e. g. cola, soda)	1	650/1,343	48.4	50.1	39.4		52.7	44.0		45.1	47.4	54.5		38.5	49.6	52.2	45.2	
	2	191/1,343	14.2	14.4	13.1		9.2	19.4		15.3	16.1	9.0		15.4	13.7	14.7	11.9	
	3	502/1,343	37.4	35.5	47.4	0.001	38.1	36.6	0.580	39.6	36.5	36.5	0.607	46.2	36.7	33.1	42.9	0.018
Calorie reduced food	1	537/1,356	39.6	40.4	35.2		34.4	44.9		42.0	41.4	32.7		30.8	31.5	39.1	46.4	
	2	430/1,356	31.7	31.4	33.3		32.5	30.9		32.5	30.3	34.0		15.4	28.1	33.9	32.0	
	3	389/1,356	28.7	28.2	31.5	0.335	33.1	24.1	0.003	25.5	28.3	33.3	0.074	61.5	40.4	27.1	21.6	<0.000

^1^How important is it for you what you eat and drink? Possible answers from 1 = not important, 2, 3, 4, 5 = very important, no answer.

^2^How important are the following facts for eating and drinking? Possible answers from 1 = not important, 2, 3, 4, 5 = very important, no answer.

^†^1 = not important (answer 1,2); 2 = moderate important (answer 3); 3 = very important (answer 4,5); no answer is not shown.

^#^Persons with answers “no answer” are not included in statistical analysis. The number of those answers can be calculated by the difference between the total population (N=1,357) and the number of answers given for each variable.

BMI, body mass index; p, p-value; data is statistically weighted by age, gender, education, domicile and BMI.

### Responsibilities for Obesity and a Healthy Diet

When asking about the general cause of a high body weight, the survey population mostly named low physical activity (82.7%, 1,120/1,354) and excessive caloric intake (80.5%, 1,079/1,341), followed by lack of will power (72.1%, 959/1,330) (**Table 4**). No statistically significant differences between the BMI groups could be found concerning the most stated variables (p > 0.05). The most stated causes of a high body weight significantly differed between education levels (p ≤ 0.01). Additionally, a significantly different percental distribution could be seen between the age groups for the variables excessive caloric intake and lack of will power (p ≤ 0.01) (**Table 4**).

**Table 4:** Reasons for a high body weight.

Variable*^1^*	Total		BMI (kg/m^2^)			Gender			Age (years)				Education (years)				
			< 30	≥ 30	p	Women	Men	p	18–35	36–65	> 65	p	Student/none	8/9	10	12/13	p
	n/N^#^	%	%	%		%	%		%	%	%		%	%	%	%	
Low physical activity	1,120/1,354	82.7	82.1	85.8	0.188	81.8	83.7	0.397	83.0	84.0	79.4	0.188	74.6	80.0	78.9	91.3	<0.000
Excessive caloric intake	1,079/1,341	80.5	79.6	84.9	0.075	80.8	80.0	0.678	82.3	84.9	68.4	<0.000	57.9	79.3	77.3	87.9	<0.000
Lack of will power	959/1,330	72.1	72.1	72.4	0.979	74.3	69.8	0.064	70.1	75.3	65.7	0.007	35.0	69.8	75.1	75.4	0.002
Big portions	908/1,316	69.0	68.7	70.6	0.614	72.0	65.9	0.015	66.2	74.2	61.0	<0.000	85.8	73.8	65.0	71.1	0.030
Medical cause	869/1,321	65.8	66.0	65.2	0.769	70.0	61.5	0.001	71.4	67.9	55.1	<0.000	45.5	69.0	64.9	66.2	0.460
Unhealthy food is cheap	847/1,332	63.6	66.0	51.4	<0.000	62.0	65.2	0.232	71.4	63.9	54.3	<0.000	66.9	54.9	65.8	70.0	<0.000
Permanent food offerings	788/1,347	58.5	59.3	54.4	0.182	58.0	59.0	0.734	57.4	60.5	55.7	0.335	64.1	57.4	53.3	64.2	0.009
Genetic reasons	705/1,325	53.2	52.7	55.8	0.445	56.0	50.4	0.040	49.9	58.2	45.7	<0.000	52.0	52.4	53.0	55.3	0.843
Advertisement/marketing for unhealthy products	666/1,345	49.5	50.9	42.3	0.015	49.0	50.1	0.720	59.5	51.4	34.2	<0.000	57.6	43.0	47.5	60.0	<0.000
Lack of knowledge about food/drink	502/1,350	37.2	38.5	30.7	0.027	38.3	36.2	0.411	44.6	39.2	24.5	<0.000	46.8	35.9	34.8	40.9	0.175
Politics	293/1,314	22.3	23.1	18.3	0.106	25.8	18.7	0.002	22.6	21.9	22.8	0.956	34.3	20.3	26.3	20.7	0.093

^1^What do you generally think is a possible cause of a high body weight? Possible answers: yes, no, no answer; Shown is “yes” as answer.

^#^Persons with answers “no answer” are not included in statistical analysis. The number of those answers can be calculated by the difference between the total population (N=1,357) and the number of answers given for each variable.

BMI, body mass index; p, p-value; data is statistically weighted by age, gender, education, domicile and BMI.

Almost 90% (89.1%, 1,197/1,343) of the survey population stated that the individual is responsible for a healthy diet, followed by the family (74.7%, 1,006/1,347) (**Table 5**). Both variables were named more often by women than men (p ≤ 0.001). Futhermore, participants with BMI ≥ 30.0 kg/m^2^ responded more often that the individual is responsible for a person´s healthy diet than participants with BMI < 30.0 kg/m^2^ (p ≤ 0.05). Additionally, statistically significant differences could be seen between the different education groups concerning the responsibility of the family for a person´s healthy diet (p ≤ 0.001) (**Table 5**).

**Table 5:** Responsibility for a person´s healthy diet.

Variable*^1^*	Answer^†^	Total		BMI (kg/m^2^)			Gender			Age (years)				Education (years)				
		n/N	^#^	< 30	≥ 30	p	Women	Men	p	18–35	36–65	> 65	p	Student/none	8/9	10	12/13	p
				%	%		%	%		%	%	%		%	%	%	%		%
Politics	1	581/1,337	43.5	43.6	43.1		41.3	45.7		46.8	42.2	42.4		46.2	43.2	46.6	38.2	
	2	295/1,337	22.1	22.0	22.0		22.0	22.1		18.0	24.8	20.4		23.1	23.0	20.1	24.3	
	3	462/1,337	34.6	34.5	35.0	0.875	36.6	32.4	0.095	35.2	33.0	37.2	0.418	30.8	34.1	33.2	37.4	0.593
Health insurance	1	473/1,340	35.3	35.6	33.4		32.9	37.7		38.4	35.5	31.2		23.1	34.0	32.0	38.8	
	2	374/1,340	27.9	28.2	26.3		28.4	27.4		28.5	29.3	24.0		7.7	24.9	30.8	29.6	
	3	493/1,340	36.8	36.1	40.3	0.221	38.5	35.0	0.175	33.1	35.1	44.9	0.003	69.3	40.8	37.0	31.3	0.001
Medicine	1	347/1,338	25.9	26.3	24.4		23.3	28.9		28.8	27.3	19.9		53.9	22.5	29.1	24.6	
	2	350/1,338	26.2	25.0	33.0		27.3	25.0		25.6	26.6	26.0		15.4	30.3	23.9	28.0	
	3	640/1,338	47.8	48.9	43.0	0.088	50.3	46.2	0.255	45.6	46.0	54.2	0.038	30.8	47.2	47.0	47.3	0.763
Media	1	360/1,342	26.8	26.4	28.7		24.0	29.7		28.2	24.8	29.5		51.8	32.4	28.0	20.8	
	2	358/1,342	26.7	26.4	28.8		26.8	26.6		22.4	31.1	21.9		0.0	27.9	26.1	27.1	
	3	623/1,342	46.4	47.2	42.5	0.223	49.2	43.6	0.038	49.4	44.1	48.2	0.216	48.2	39.9	46.4	52.0	0.006
Food industry	1	240/1,338	17.9	17.9	18.7		14.3	21.6		17.2	15.2	24.6		27.9	18.8	20.9	11.7	
	2	256/1,338	19.1	19.1	19.2		19.5	18.9		21.3	19.9	15.2		8.0	19.4	19.7	20.4	
	3	840/1,338	62.8	63.0	61.7	0.796	66.2	59.4	0.010	61.5	64.8	59.7	0.259	64.1	61.8	59.3	68.2	0.043
Family	1	115/1,347	8.5	8.2	10.3		6.7	10.4		5.9	8.4	11.7		7.7	8.9	8.6	6.0	
	2	227/1,347	16.9	16.2	20.2		14.6	19.2		19.1	15.9	16.1		38.5	25.1	15.5	11.4	
	3	1,006/1,347	74.7	75.6	70.0	0.079	78.8	70.4	<0.000	74.9	75.5	72.2	0.490	53.8	66.3	76.0	82.3	<0.000
The individual	1	32/1,343	2.4	2.4	2.0		1.9	2.9		2.9	1.75	9.8		0.0	2.7	3.1	1.3	
	2	113/1,343	8.4	9.3	3.9		5.3	11.6		6.0	9.5	5.0		0.0	9.4	7.8	6.1	
	3	1,197/1,343	89.1	88.3	94.1	0.011	92.8	85.5	<0.000	90.0	88.8	84.6	0.845	100.0	87.9	89.1	92.6	0.068
Cafeteria	1	247/1,282	19.3	19.8	16.5		20.4	17.9		15.5	18.6	24.6		18.1	23.3	19.8	13.7	
	2	325/1,282	25.4	25.1	26.4		22.6	28.3		25.8	27.5	20.1		0.0	23.4	26.5	25.8	
	3	711/1,282	55.5	55.1	57.0	0.514	57.0	53.8	0.268	58.7	53.8	55.3	0.344	81.8	53.0	53.8	60.2	0.031
School	1	213/1,291	16.4	16.3	18.1		14.1	19.0		17.1	14.0	21.0		38.5	17.1	15.0	13.1	
	2	319/1,291	24.7	24.9	23.8		25.1	24.1		22.4	28.2	19.2		0.0	24.3	24.6	27.6	
	3	759/1,291	58.9	58.9	58.5	0.902	60.6	57.1	0.175	60.5	57.7	59.4	0.682	69.3	58.6	60.6	59.1	0.914

^1^In your opinion, who is responsible for a person`s healthy diet? Possible answers from 1 = no responsibility, 2, 3, 4, 5 = high responsibility, do not know, no answer.

^†^1 = no responsibility (answer 1,2); 2 = moderate responsibility (answer 3); 3 = high responsibility (answer 4,5); do not know and no answer are not shown.

^#^Persons with answers “no answer” or “do not know” are not included in statistical analysis. The number of those answers can be calculated by the difference between the total population (N=1,357) and the number of answers given for each variable.

BMI, body mass index; p, p-value; data is statistically weighted by age, gender, education, domicile and BMI.

Most participants responded to prefer most promoting regional and seasonal foods (82.8%, 1,099/1,327), nutrition education in school (86.1%, 1,148/1,333) and implementation of a healthy diet in the kindergarten (88.4%, 1,185/1,340) when the policy should take care of a person´s healthy diet (**Table 6**). The taxation of specific foods was observed to be the least preferred political approach (41.6%, 550/1,323) (**Table 6**). No statistically significant differences between BMI groups could be observed except for one political approach. Participants with BMI < 30.0 kg/m^2^ answered more often to prefer political approaches supporting a healthy diet by promoting regional and seasonal foods than participants with BMI ≥ 30.0 kg/m^2^ (p ≤ 0.05). Women showed a higher willingness to support political approaches for a healthy diet than men, depending on the kind of political regulatory action (p ≤ 0.05). The percental distribution of most of the political approaches was significantly different between the age groups (p ≤ 0.01). Similar results could be found when comparing the different education groups (p ≤ 0.01).

**Table 6:** Opinions of participants about regulatory and other measures to achieve a healthy diet in the population.

Variable*^1^*	Answer^†^	Total		BMI (kg/m^2^)			Gender				Age (years)				Education (years)				
		n/N^#^	%	< 30	≥ 30	p	Women		Men	p	18–35	36–65	> 65	p	Student/none	8/9	10	12/13	p
				%	%			%	%		%	%	%		%	%	%	%	
Taxation of specific foods (e.g. sugar tax)	1	498/1,323	37.6	36.8	42.5			38.3	36.9		43.8	36.9	32.5		69.2	41.4	42.7	30.1	
	2	275/1,323	20.8	22.2	13.7			20.8	20.8		23.8	21.4	16.1		0.0	19.8	16.5	24.4	
	3	550/1,323	41.6	41.0	44.8	0.355		40.8	42.4	0.573	32.4	41.8	50.8	<0.000	30.8	38.8	40.8	45.4	0.169
Traffic light labelling (e.g. red for unhealthy food)	1	225/1,320	17.0	17.3	15.5			16.7	17.4		22.1	15.6	14.6		46.2	12.2	18.7	16.9	
	2	203/1,320	15.4	16.0	12.2			14.4	16.4		19.5	16.8	7.8		7.7	17.6	12.7	14.4	
	3	892/1,320	67.6	66.6	71.8	0.121		68.9	66.4	0.301	58.4	67.7	77.3	<0.000	46.2	70.5	68.7	68.4	0.301
Lowering of prices for healthy foods	1	195/1,317	14.8	15.0	13.7			13.7	16.0		11.8	16.8	13.6		27.8	17.1	10.2	17.4	
	2	204/1,317	15.5	15.4	16.0			16.1	15.0		19.1	15.6	11.2		0.0	9.2	14.6	22.1	
	3	918/1,317	69.7	69.5	70.3	0.895		70.3	69.1	0.680	72.1	67.6	74.7	0.074	72.2	73.4	75.2	60.3	<0.000
Increasing of prices for unhealthy foods	1	373/1,322	28.2	28.5	26.9			27.5	29.0		30.9	27.9	26.1		46.2	26.1	32.9	25.1	
	2	295/1,322	22.3	22.3	22.6			21.4	23.2		26.4	23.1	16.3		7.7	24.4	17.4	25.3	
	3	653/1,322	49.4	49.3	50.0	0.800		51.0	47.8	0.236	42.7	49.0	57.5	0.001	46.2	49.3	49.6	49.5	1.000
Ban of advertising for unhealthy foods	1	353/1,305	27.0	26.6	30.1			24.5	29.7		33.7	25.9	22.3		53.8	26.4	31.6	22.3	
	2	271/1,305	20.8	21.1	19.1			20.4	21.2		30.4	20.4	10.6		15.4	11.8	19.8	28.9	
	3	681/1,305	52.2	52.5	51.2	0.687		55.3	49.1	0.026	35.9	53.5	67.1	<0.000	30.8	61.8	48.3	48.1	<0.000
Improving food quality (e.g. less sugar)	1	128/1,330	9.6	9.9	8.5			8.8	10.7		12.4	8.8	8.4		53.8	7.8	11.6	7.6	
	2	160/1,330	12.0	12.6	9.0			9.1	15.1		14.7	12.0	9.0		7.7	8.9	11.6	13.6	
	3	1,042/1,330	78.3	77.5	83.0	0.098		82.2	74.4	0.001	72.9	79.0	82.3	0.009	38.5	83.2	76.8	78.8	<0.000
Awareness campaign	1	117/1,333	8.8	8.8	8.5			7.4	10.3		14.5	7.4	5.4		46.2	7.6	12.4	6.3	
	2	202/1,333	15.2	14.3	19.8			13.4	16.9		19.8	13.7	13.1		15.4	16.3	12.8	15.8	
	3	1,014/1,333	76.1	76.8	71.7	0.099		79.3	72.8	0.005	65.8	78.7	80.8	<0.000	38.5	76.1	74.8	77.8	0.009
Promoting regional and seasonal foods	1	84/1,327	6.3	5.8	9.0			4.6	8.1		7.4	6.4	5.2		46.2	7.0	5.5	5.7	
	2	143/1,327	10.8	10.3	13.7			8.1	13.5		13.1	10.5	9.0		7.7	12.6	5.8	10.0	
	3	1,099/1,327	82.8	83.8	77.8	0.030		87.1	78.4	<0.000	79.5	83.0	85.7	0.112	46.2	80.4	86.6	84.0	<0.000
Nutrition education in school	1	56/1,333	4.2	4.5	2.4			4.2	4.2		5.3	3.9	3.6		15.4	4.2	3.3	4.9	
	2	129/1,333	9.7	9.8	8.5			7.7	11.7		16.8	8.0	5.5		30.8	10.7	8.7	8.9	
	3	1,148/1,333	86.1	85.5	89.2	0.199		88.1	83.9	0.027	77.9	88.0	90.0	<0.000	53.8	85.1	88.0	86.1	0.003
Promoting a healthy diet in kindergarten	1	73/1,340	5.4	5.5	4.7			4.7	6.2		8.7	4.7	3.5		46.2	6.1	3.5	4.8	
	2	83/1,340	6.2	6.6	4.3			6.3	6.1		11.6	4.2	4.5		7.7	6.4	4.0	6.3	
	3	1,185/1,340	88.4	88.0	91.0	0.242		88.8	87.9	0.521	79.7	91.1	92.0	<0.000	46.2	87.2	92.2	88.9	<0.000

^1^When the policy should take care of a person`s healthy diet which of the following approaches would you personally prefer? Possible answers from 1 = I disclaim, 2, 3 = I support, do not know, no answer.

^†^1 = no support (answer 1); 2 = moderate support (answer 2); 3 = high support (answer 3); do not know and no answer are not shown.

^#^Persons with answers “no answer” or “do not know” are not included in statistical analysis. The number of those answers can be calculated by the difference between the total population (N=1,357) and the number of answers given for each variable.

BMI, body mass index; p, p-value; data is statistically weighted by age, gender, education, domicile and BMI.

## Discussion

This survey provided representative data on the general population´s opinion in Germany about who is responsible for the development of obesity and for promoting a healthy diet. Over 80% of the survey population stated that excessive caloric intake and low physical activity are causes of a high body weight. Furthermore, over 70% of participants indicated that a lack of will power is another cause for a high body weight. In their recent survey, Kim et al. (21) compared the obesity stigma between Germany and the US. In the German survey, a person with obesity was assigned the following attributes: poor self-control, no will power, self-indulgent, inactive, shapeless, slow, unattractive, lacking endurance, overeating, and liking food (21). In summary, becoming obese was seen as a general loss of self-control. Likewise, almost 90% of this survey population thought that the responsibility for adhering to a healthy diet lies with the individual. Moreover, persons with BMI ≥ 30 kg/m^2^ named the individual´s responsibility towards a healthy diet more often than those with BMI < 30 kg/m^2^.

Obesity is recognized as a complex chronic disease in the scientific community (15). The judgment of self-blaming, described above, which is often ascribed to individuals with obesity may represent a general misunderstanding and underestimation of the genetic influence on the regulation of body weight and the impact of external drivers. Indeed, a broad misunderstanding may explain, at least in part, the high prevalence of weight-related stigma in the population and society in general. People with obesity are confronted with weight-related stigma through the media (22, 23), the health care system (24, 25), the workplace (25), educational settings, and friends and family (25, 26). Several studies pointed out that weight-related stigma can lead to negative outcomes, e.g. increased physiological dysfunction (27), as well as decreased cardiovascular (28) and mental health (12, 14). Moreover, weight-related stigma is associated with increased weight and waist circumference (13, 29) and increased risk of becoming overweight (29). This might be due to a higher consumption of food, a confirmed association between weight-related stigma and binge eating (30), and decreased motivation for physical activity (31). The latter may be explained by the fact that people with obesity tend to avoid places in which weight-related stigma occurs (31). Thereby, a vicious cycle may occur, resulting from a combination of stigmatization from oneself as well as from others. It is essential that the society as a whole, including affected persons, experts, media, stakeholders and the general public, recognizes that obesity is a chronic disease that is impacted by e.g. genetic, physiological, psychosocial and environmental factors. Ultimately, this shift in perspective may contribute to improved preventive and therapeutic strategies.

Approaches for the prevention and treatment of obesity on an individual level have shown little effect (32). As the development of obesity is influenced through micro- as well as through macro-settings (32), several strategies focusing on these settings were established (32, 33). The WHO indicated that food choices are influences by several determinants, e.g. price and availability. Moreover, advertisement of unhealthy food promotes poor food choices among children and adolescents (32). In their strategy paper for primary prevention, the German Alliance against NCDs stated four approaches which focused on the macro level: increasing physical activity among children and adolescents (primarily in school), food taxation, healthier food in kindergarten and school, and banning advertisement for unhealthy food and beverages (33). A systematic review focusing on the effects of environmental interventions on the consumption of sugar-sweetened beverages found evidence that several strategies are associated with a reduced consumption of sugar-sweetened beverages. In particular, they observed that food labeling and increased prices led to reduced consumption of sugar-sweetened beverages (34). Over 70% of the survey population presented herein indicated that they would support the following political actions: traffic light labelling, lowering of prices for healthy food, improving food quality, awareness campaigns, promoting regional and seasonal foods, nutrition education in school and promoting a healthy diet in kindergarten. Surprisingly, increasing prices and banning the advertisement of unhealthy food was named as appropriate strategies by 50% of this survey population. This suggests that half of the respondents, independent of the BMI, are generally accepting political interventions to reduce an obesogenic environment. A recently published German survey with 1,035 persons found similar results (35). About 64% of the participants were in favor for political strategies concerning healthier food. Furthermore, Jürkenbeck et al. (35) has shown that the support of political strategies was independent of the struggle of choosing healthy food.

Our data showed that the most frequently used strategies for weight reduction were increased physical activity, a calorie-reduced diet, a low-carb diet, and a low-fat diet. Adherence to a weight loss program, either at a medical center or a commercially available program such as Weight Watchers^®^, weight loss medications or surgery were reported as weight loss strategies by less than 10% of the survey population. Persons with a BMI ≥ 30 kg/m^2^ named professional programs or the intake of medication more often than those with a BMI < 30 kg/m^2^. Our results suggest that people with overweight or obesity are more likely to apply self-selected methods to losing weight rather than following evidence-based therapies. Furthermore, these results indicate that people with overweight or obesity do not get the help they needed and have to deal with their weight problems on their own. However, as the survey population was not asked whether they decided on the weight loss strategy on their own or whether the strategy was selected or performed by a nutritional specialist this is just an assumption. Continuously given advice for a healthy lifestyle in the media (22) and the common opinion indicating that individuals are in charge of their healthy lifestyle (21) may explain why professional support is only rarely used. This is in line with the results of a survey with 14,502 people with obesity and 2,785 healthcare professionals (36). Caterson et al. (36) found out, that 81% of the participants with obesity stated that losing weight is their own responsibility. Only 26% of those named their healthcare professional as responsible for a successful weight loss (36). This is supported by the fact that the treatment of obesity is not covered by health care systems and costs have to be paid by the patients themselves. Therefore, it is crucial to raise awareness of obesity as a chronic disease both in the general population and among stakeholders.

When asked about personal eating and drinking behaviors, almost 80% of the survey population stated that eating and drinking is important for them. Due to the question asked it is not possible to specify this result. Self-prepared and fresh meals, adequate fluids, healthy and tasty foods are important factors for the selection of eating and drinking offerings. However, the comparison of persons with and without obesity showed different results. For people with BMI ≥ 30 kg/m^2^, eating and drinking seemed to be less important than for those without obesity. Furthermore, factors like fiber intake, balanced and healthy food, fruit and vegetable intake and vegetarian/vegan food were less stated to be important for those with BMI ≥ 30 kg/m^2^ than for those with BMI < 30 kg/m^2^. This is in line with the other results observed. Persons with BMI ≥ 30 kg/m^2^ blame themselves for being personally responsible for their high body weight by choosing a less healthy diet. Moreover, having a lack of knowledge about food and drink was stated significantly less by persons with BMI ≥ 30 kg/m^2^ than those with BMI < 30 kg/m^2^. This might indicate, that even though most persons with obesity have knowledge about a healthy diet, the negative effects of self-blaming and weight-related stigma hold up the vicious circle and prevent persons with obesity from living a healthy lifestyle. Therefore, reducing weight-related stigma and increasing the knowledge of obesity to be a chronic disease is crucial for the prevention and treatment of obesity.

This survey provides data on the opinion of a representative and rather large sample regarding the responsibility of stakeholders for obesity and a healthy diet in Germany. The study-specific questionnaire was developed by experts and the interviews were conducted in a standardized manner (CATI method) by a professional agency. However, several limitations should be mentioned.

The anthropometric data for the BMI calculation was obtained by self-report. However, this has been accepted as a valid method (37, 38). Although the systematic short screening of additional individuals to have more participants with BMI ≥ 30.0 kg/m^2^ might have methodological limitations, the standardized use of an RDD sampling method and the statistical weighting of data (according to age, gender, education, domicile, and BMI) produced representative data for adults in Germany. However, this data is also biased by participation of people who are motivated for surveys and interested in the given topic. In addition, it has to be mentioned that this survey is focused on diet as one of the main factors associated with overweight and obesity. It might be of added value to extend the results by parameters of physical activity. Besides, data on personal health determinants such as self-efficacy would enrich the findings. As most of the questions asked were close-ended, the results are limited to given answers and might be biased to give a certain self-selected response. However, the survey is strengthened by the fact, that the different answer options within one question were randomly chosen for each participant to avoid order bias.

Based on the results of this survey and the findings of the policymaker survey from EASO (16) the following implications may be derived. First, an increased awareness that obesity is a chronic disease and not self-inflicted is necessary for a more successful strategy to prevent and to treat obesity. Educating the public that obesity is a multifactorial disease is key to reducing stigmatization and discrimination. Second, policymakers should understand their role in setting and implementing a public health agenda that promotes a healthy lifestyle, reduces an obesogenic environment, and provides better access to services for those who struggle with obesity. Finally, healthcare professionals should be given the tools and education to allow them to adequately manage and support their patients with obesity, including addressing the complex nature of obesity as a medical condition. The findings from this survey support the need for a multidisciplinary approach from all members of society to tackle the obesity epidemic (39) and to reframe obesity as a chronic disease that requires individual, societal and political engagement to plan better prevention and treatment strategies.

## Conclusions

In this survey, the opinions of the general population on the responsibilities of individuals and stakeholders for obesity and a healthy diet were assessed. Most of the survey participants indicated that obesity is caused by self-controlled attitudes and individuals are personally responsible for a healthy diet. These beliefs may promote the development of a weight-related stigma in the population. Hence, more education and communication concerning the true and complex causes of obesity are needed to reduce weight-related stigma. Furthermore, the survey population revealed a high acceptance of political approaches to facilitate a healthy diet. Therefore, the time has come to address the obesogenic environment to promote and achieve a healthier lifestyle in the general population.

## Data Availability Statement

The datasets generated for this study are available on request to the corresponding author.

## Ethics Statement

Ethics committee approval: Ethical review and approval was not required for the study on human participants in accordance with the local legislation and institutional requirements.

Consent procedures: Written informed consent from the participants was not required to participate in this study in accordance with the national legislation and the institutional requirements. Participants provided their oral informed consent prior to participation, under the premise that participation in this survey was voluntary and analyses are conducted anonymously. Oral consent is common in representative survey research in Germany in order to avoid bias through non-response.

## Author Contributions****

SB analyzed the data and wrote the manuscript. TD designed the survey. GS analyzed the data. HH commented on the manuscript. CH designed the survey, analyzed data and wrote the manuscript. All authors contributed to the article and approved the submitted version.

## Funding****

This article was supported by a grant of the German Federal Ministry of Education and Research (Bundesministerium für Bildung und Forschung, BMBF, funding code: 01EA1709, *enable* publication number: 051). This article was written by the Junior Research Group “Personalized Nutrition & eHealth” within the Nutrition Cluster *enable*. This work was also supported by the German Research Foundation (DFG) and the Technical University of Munich (TUM) in the framework of the Open Access Publishing Program.

## Conflict of Interest****

CH is a member of the scientific advisory board of 4sigma GmbH (Oberhaching, Germany). HH is a member of the scientific advisory board of the Almeda GmbH (Munich, Germany) and the Oviva AG (Zurich, Switzerland).

The remaining authors declare that the research was conducted in the absence of any commercial or financial relationships that could be construed as a potential conflict of interest.
